# Clinical characteristics associated with bone mineral density improvement after 1-year alendronate/vitamin d_3_ or calcitriol treatment: Exploratory results from a phase 3, randomized, controlled trial on postmenopausal osteoporotic women in China

**DOI:** 10.1097/MD.0000000000011694

**Published:** 2018-08-03

**Authors:** Er-Yuan Liao, Zhen-Lin Zhang, Wei-Bo Xia, Hua Lin, Qun Cheng, Li Wang, Yong-Qiang Hao, De-Cai Chen, Hai Tang, Yong-De Peng, Li You, Liang He, Zhao-Heng Hu, Chun-Li Song, Fang Wei, Jue Wang, Lei Zhang

**Affiliations:** a The Second Xiangya Hospital, Central South University, Changsha; b The Sixth People's Hospital, Shanghai Jiaotong University; c Peking Union Medical College Hospital; d Nanjing Drum Tower Hospital, Nanjing; e Huadong Hospital Affiliated to Fudan University; f Tianjin Hospital, Tianjin; g The Ninth People's Hospital; h West China Hospital, West China School of Medicine, Sichuan University, Chengdu; i Beijing Friendship Hospital, Capital Medical University; j The First People's Hospital; k Beijing Jishuitan Hospital; l Peking University People's Hospital; m Peking University Third Hospital, Beijing; n Global Medical Affairs, Merck Sharp & Dohme China, Shanghai, China.

**Keywords:** alendronate sodium, bone mineral density, bone turnover biomarker, calcitriol, post hoc analysis, postmenopausal osteoporosis

## Abstract

Baseline and on-treatment characteristics, including age, obesity, calcium intake, and bone turnover markers, may predict the bone mineral density (BMD) response in women with postmenopausal osteoporosis (PMO) to 1 to 2 years of antiresorptive therapy and/or vitamin D supplementation. This study aimed to explore clinical characteristics associated with 12-month BMD improvement in Chinese women with postmenopausal osteoporosis (PMO).

In this post hoc analysis of a previous phase 3 multicenter, randomized controlled trial, Chinese PMO women who were treated with once weekly alendronate 70 mg/vitamin D3 5600 IU (ALN/D5600) or once daily calcitriol 0.25 mcg, and had measurements of 1-year lumbar spine BMD (LS-BMD) and on-treatment bone turnover markers (BTMs) were included in the analysis.

In Chinese PMO patients on ALN/D5600, 1-year LS-BMD change was negatively correlated with age (*β* = −0.00084, *P* < .01), dietary calcium (*β* = −0.0017, *P* = .07), and procollagen type 1 N-terminal propeptide (P1NP) change at month 6 (*β* = −0.000469, *P* = .0016), but positively with body mass index (BMI) (*β* = 0.00128, *P* = .08); baseline P1NP above the median was associated with a significantly greater BMD percentage change at the lumbar spine (*P* = .02) and the total hip (*P* = .0001). In the calcitriol group, a significant 1-year LS-BMD increase was associated with BMI (*β* = 0.0023, *P* = .02), baseline P1NP (*β* = 0.00035, *P* = .0067), history of prior vertebral fracture(s) (*β* = 0.034, *P* < .0001) and baseline serum 25(OH)D level (*β* = −0.00083, *P* = .02).

The presented findings from Chinese postmenopausal osteoporotic women suggested clinically meaningful baseline and on-treatment characteristics predicting BMD improvement after 1 year of ALN/D5600 treatment, which differed from calcitriol treatment with baseline identifiable associations. The study remained exploratory and further accumulation of evidence is needed.

## Abstract 2

Supplemental Digital Content is available in the text

## 1 Introduction

Osteoporosis, a major public health concern worldwide, manifests as loss of bone minerals and consequently increases the risks of fall and fracture.^[1]^ Many risk factors have been established for osteoporosis, mainly including age, female sex, estrogen deficiency, and ethnicity, all of which put postmenopausal patients at a higher risk for osteoporosis.^[2]^ Osteoporosis also prevails in Chinese postmenopausal women, with a prevalence of approximately 40.1% in a national registry study.^[3]^ Moreover, vitamin D deficiency is a common health concern in Chinese women, with up to 80% of women aged 40 to 75 years residing in northwestern China afflicted with vitamin D insufficiency (25-hydroxyvitamin D [25(OH)D] < 20 ng/mL), a debatable risk factor for osteoporosis depending on dietary intake.^[4]^ Supplementary vitamin D combined with calcium, however, has been well accepted for prevention and treatment of postmenopausal osteoporosis.^[5]^

As recommended by the American and British guidelines, use of oral bisphosphonates has become the first-line treatment modality for postmenopausal osteoporosis due to a documented preventative effect on fracture at multiple sites through increased bone mineral density (BMD).^[6–8]^ A previous randomized, open label, controlled study demonstrated in Chinese postmenopausal women with osteoporosis that weekly alendronate 70 mg/vitamin D_3_ 5600 IU (ALN/D5600) resulted in a significantly greater increase in lumbar spine BMD (LS-BMD) and a greater reduction in bone turnover marker (BTM) measurements than daily 0.25 μg calcitriol alone within 1 year, although the benefit regarding the reduction in fracture risk remains to be investigated.^[9]^

Vitamin D receptor polymorphism is well known to be a major factor predictive of BMD response after calcitriol treatment,^[10]^ and a number of studies also reported that the short-term change in BTM measurements can predict the BMD response to bisphosphonate treatment in Caucasian and Eastern Asian postmenopausal osteoporosis patients.^[11,12]^ There is a knowledge gap regarding which baseline and on-treatment characteristics can identify subgroups of Chinese women who will benefit more from antiresorptive therapy with respect to BMD improvement. Based on previously reported randomized controlled study (RCT), it was hypothesized that patient's clinical characteristics may be significantly associated with BMD improvement after 1-year antiresorptive treatment and thus impact therapeutic approach in clinical practice. This post hoc analysis aimed to explore the demographic and clinical characteristics associated with the 1-year LS-BMD change among Chinese postmenopausal osteoporosis patients who were previously randomized to receive 1-year ALN/D5600 or calcitriol. Furthermore, this exploratory analysis investigated baseline patient and on-treatment characteristics, especially BTM measurements and vitamin D status, associated with increases in BMD.

## 2 Patients and methods

This study was approved by the independent ethics committees of 13 participating sites. A written informed consent was obtained in accordance with the principles and provisions of the International Conference on Harmonization-E6 Guideline for Good Clinical Practice, following country-specific consent requirements.

The design and results of the alendronate/vitamin D3 study in Chinese postmenopausal women have been published (clinicaltrials.gov number NCT01350934).^[9]^ This was a 12-month randomized, open-label, active-comparator-controlled study evaluating the efficacy and safety of ALN/D5600 once weekly as a combination tablet versus those of calcitriol 0.25 μg daily in the treatment of osteoporosis in postmenopausal women in China. Briefly, 219 women Chinese osteoporosis patients, who were aged >55 years and postmenopausal for at least 1 year, were randomized to receive 1-year ALN/D5600 (n = 111; Frosst Iberica SA, Madrid, Spain) or calcitriol alone (n = 108; R.P. Scherer GmbH & Co. KG, Eberbach Baden, Germany). The patients were also required to have the following: BMD T-score ≤–2.5 in at least 1 anatomic site (LS, total hip, or femoral neck) or prior non-pathological fragility fracture (of the spine, wrist, humerus, or clavicle) and BMD T-score ≤–1.5 in at least 1 of the same anatomic sites; 25(OH)D levels ≥8 ng/mL (20 nmol/L); no prior hip fracture; and no abnormal finding of clinical significance. The study was sponsored by MSD China Holding Co., Ltd., China, and jointly administered with PAREXEL China. BMD was assessed at the time of screening, month 6, and month 12 using dual-energy x-ray absorptiometry, to calculate the percentage change from the baseline for the LS, total hip, femoral neck, and trochanter; lateral spine x-ray of T4 to L5 (1 thoracic and 1 lumbar x-ray) was also performed at the time of screening, month 6, and month 12. Biochemical markers of bone turnover, including procollagen type 1 N-terminal propeptide (P1NP) and serum C-terminal telopeptide (s-CTx), were measured at baseline, month 6, and month 12 by an independent, centralized laboratory (Quest Diagnostics, Shanghai, China) using an immunoassay and electrochemiluminescent immunoassay (Roche Cobas E601 module immunology analyzer; Roche Diagnostics, Indianapolis, IN), respectively. Plasma 25(OH)D levels were also assessed and monitored by the central laboratory using liquid chromatography-tandem mass spectrometry throughout the study period. Vitamin D insufficiency was defined as plasma 25(OH)D <20 or 15 ng/mL.

### 2.1 Statistical analyses

The present study was designed as a post-hoc exploratory analysis of a previous phase 3 randomized controlled trial comparing ALN/D5600 with calcitriol for BMD improvement in Chinese postmenopausal women. All patients (n = 96) with on-treatment BTM (P1NP/s-CTx) and 1-year LS-BMD measurements were eligible for analysis. The phase 3 RCT had statistically 97% power (2-sided, *α* = 0.05) to demonstrate the primary hypothesis that treatment with ALN/D5600 was superior to treatment with calcitriol assuming that the treatment difference in BMD percent change between ALN/D5600 IU and calcitriol is 2%. Because the current exploratory analysis had no priori statistical hypothesis, the sample size used for the study was based on the actual number of subjects included. For the multivariate analysis model used in the study, power calculation was performed as the study team anticipated that there could be 4 to 6 independent variables (clinical characteristics) included for the prediction of the dependent variable (BMD improvement). Assuming an *R*^2^ between 0.1 and 0.3 (moderate-to-good fitness to the regression model) and 4 to 6 variables entered into the model, the estimated statistical power for the multiple regression model was between 65% and 99%.

Baseline characteristics (age, body weight, body mass index [BMI], LS-BMD, prior vertebral fracture, dietary calcium intake, serum 25(OH)D, BTMs) and BTM changes at month 6 were selected for univariate analysis based on clinical discretion; for baseline BTMs, P1NP and s-CTx, the log scale was the analytic metrics to force the normal distribution to be analyzed in the model. Continuous variables with a *P*-value <.25 in a simple linear regression or a general linear model were included in multivariate analysis. A multiple regression model by the least squares method was constructed to explore variables significantly correlated with 1-year LS-BMD change. Hierarchical forward selection with switching was applied for variable elimination until all variables remaining in the model had a *P*-value <.1 for a standardized coefficient (*β*). In addition, a constrained longitudinal data analysis method with unstructured covariance matrix is used to model the correlation among repeated measurements. The model includes percent change of BMD from baseline as response variable, and includes terms for time, 25(OH)D (baseline, month 12, tertiles) or BTMs (baseline, month 12, median), the interaction of time by tertiles of baseline 25(OH)D. All statistical analyses were performed using the Statistical Analysis System 9.4 (SAS Institute, Cary, NC).

## 3 Results

### 3.1 Patients

In previously reported RCT, the 2 treatment groups had comparable demographic and baseline characteristics.^[9]^ After 12-month treatment, percentage increase in LS-BMD was significantly higher in the ALN/D5600 group. On-treatment BTMs and proportion of vitamin D insufficiency reduced significantly in the ALN/D5600 group, but remained slighted improved in the calcitriol group. According to the predefined eligibility criteria, data from 200 subjects in the RCT were included in the post hoc analysis (ALN/D5600 = 96, calcitriol = 104).

### 3.2 Characteristics associated with LS-BMD improvement after ALN/D5600 treatment

As predefined, 96 patients in the ALN/D5600 group were eligible for analysis, with a mean increase in LS-BMD of 0.03 g/cm^2^ at 1 year. Baseline characteristics, including age, body weight, BMI, dietary calcium intake, baseline levels of serum BTMs and 25(OH)D, and on-treatment changes in BTMs at month 6 from the baseline showed a significant association with the 1-year LS-BMD on the univariate analysis (all *P* < .25) and were entered into the multivariate model (Table 1). On multivariate analysis, the 1-year LS-BMD increase was negatively associated with age (*β* = −0.00084, *P* < .01), dietary calcium (*β* = −0.0017, *P* = .07), and P1NP change at month 6 (*β* = −0.000469, *P* = .0016) but positively associated with BMI (*β* = 0.00128, *P* = .08).

**Table 1:**
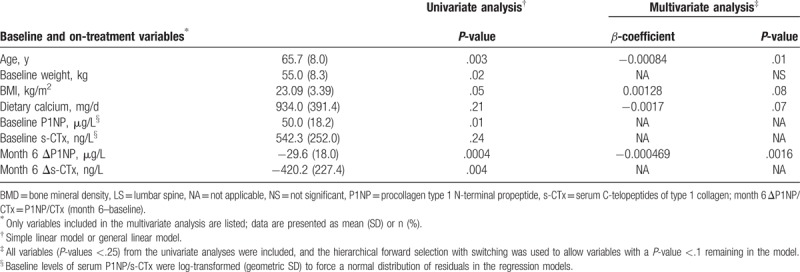
Linear modeling of characteristics associated with 1-year LS-BMD improvement in patients on ALN/D5600 (n = 96).

**Figure 1:**
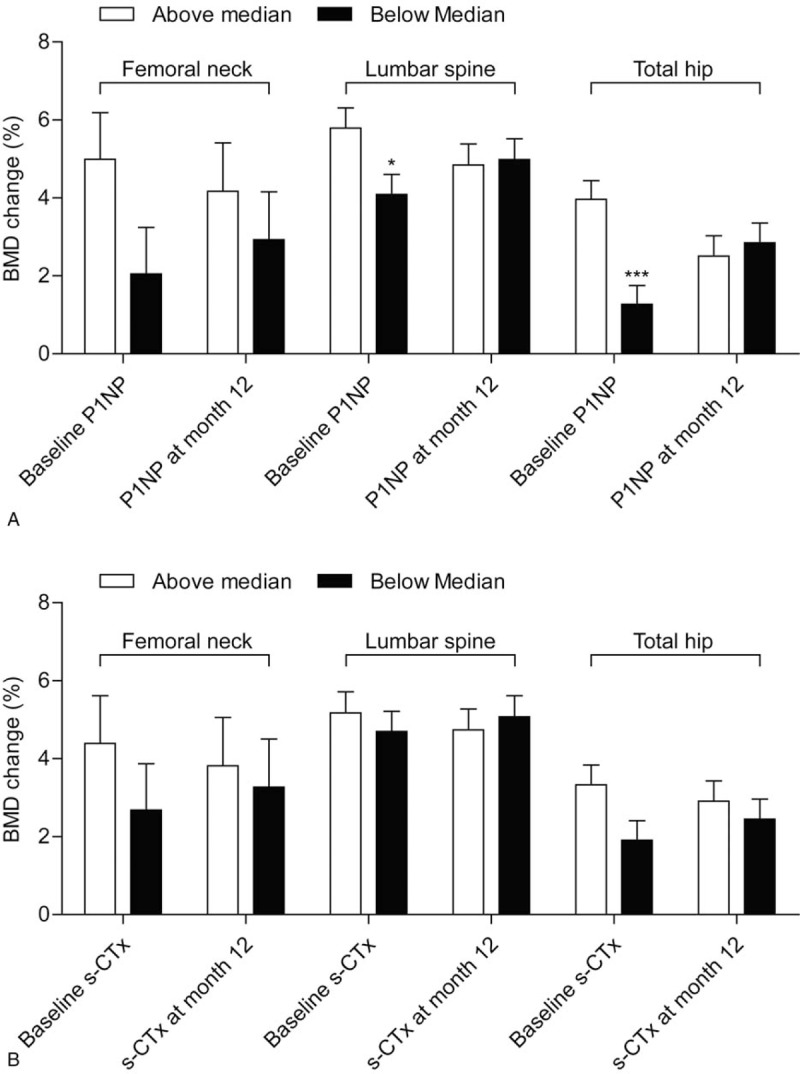
Correlation of 12-month mean BMD percentage change at the lumbar spine, femoral neck, and total hip with tertiles of BTMs at the baseline and 12 months in patients treated with 12-month ALN/D5600. A longitudinal data analysis with the unstructured covariance matrix was used to model the correlation among repeated measurements. The model includes the percent change in BMD from the baseline as a response variable and includes terms for time, baseline P1NP, or s-CTx stratum (≤median vs >median), and the interaction of time by baseline P1NP or s-CTx stratum (≤median vs >median). Error bars represent standard error. BMD = bone mineral density, BTM = bone turnover marker, P1NP = procollagen type 1 N-terminal propeptide, s-CTx = serum C-terminal telopeptide.

Baseline median levels of P1NP and s-CTx were 47.1 μg/L and 533 ng/L, respectively. At 12 months, LS-BMD changes were 5.79 ± 0.52% for above median P1NP (n = 48) and 4.09 ± 0.51% for below median P1NP (n = 49); and 5.18 ± 0.54% for above median s-CTx (n = 47) and 4.70 ± 0.52% for below median s-CTx (n = 50) (Fig. 1). Patients with a baseline P1NP level above the median had a significantly greater 12-month BMD increase at the LS, femoral neck, and total hip than those with levels below the median (all *P* < .05); however, those with P1NP above and below the median at month 12 had a similar BMD increase at these 3 anatomic sites (all *P* > .05). In contrast, patients with a baseline s-CTx above or below the median at the baseline and month 12 had a similar BMD increase at all 3 anatomic sites (all *P* > .05). Results for BMD percent change correlated with BTMs are available in Supplementary Table 1.

**Figure 2:**
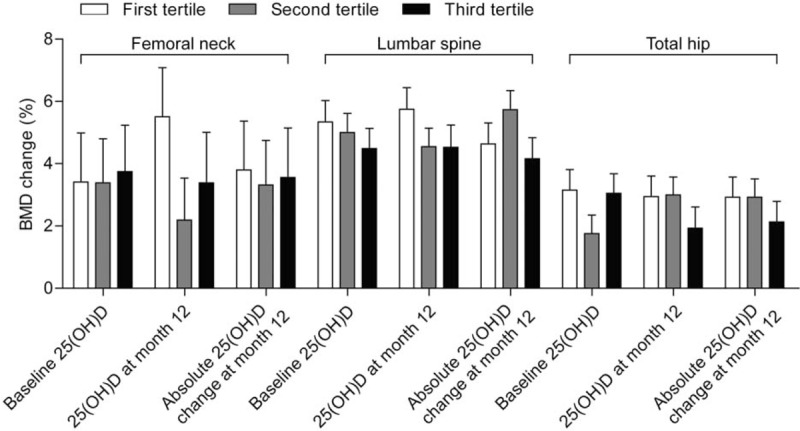
Correlation of 12-month mean BMD percentage change at the lumbar spine, femoral neck, and total hip with tertiles of plasma 25(OH)D at the baseline and 12 months and with absolute change in patients treated with 12-month ALN/D5600. A longitudinal data analysis with the unstructured covariance matrix was used to model the correlation among repeated measurements. The model includes percent change in BMD from the baseline as a response variable and includes terms for time, tertiles of baseline 25(OH)D, and the interaction of time by tertiles of baseline 25(OH)D. Error bars represent standard error. BMD = bone mineral density.

The baseline 25(OH)D tertile cut-offs were 16 and 23 ng/mL, respectively. At month 12, LS-BMD percent changes were 5.34 ± 0.69% in the 1st tertile, (n = 29), 5.00 ± 0.62% in the 2nd tertile (n = 36), and 4.49 ± 0.65% in the 3rd tertile (n = 32) (Fig. 2). None of the baseline, 12-month, or absolute changes within 12 months in the tertiles of plasma 25(OH)D level was associated with 12-month BMD percentage increases at the LS, femoral neck, or total hip (Fig. 2). Post hoc repeated measures analysis of BMD percent changes showed no statistical significance for all comparisons between baseline 25(OH)D tertiles (all *P* < .05) at the LS, femoral neck, and total hip. Results for BMD percent change correlated with 25(OH)D are available in Supplementary Table 2.

### 3.3 Characteristics associated with LS-BMD improvement after calcitriol treatment

As predefined, 104 patients in the calcitriol group were eligible for analysis, with a mean increase in LS-BMD of 0.01 g/cm^2^at 1 year. Baseline characteristics, including age, BMI, LS-BMD, BTMs, on-treatment s-CTx change at month 6, prior vertebral fracture(s), and serum 25(OH)D were significantly associated with 1-year LS-BMD improvement (all *P* < .25) and further analyzed in the multivariate model (Table 2). In contrast, all the aforementioned characteristics analyzed in the univariate model showed no significant association with the 1-year LS-BMD increase (all *P* > .1), except for BMI (*β* = 0.0023, *P* = .02), baseline P1NP (*β* = 0.00035, *P* = .0067), prior vertebral fracture(s) (*β* = 0.034, *P* < .0001), and baseline serum 25(OH)D level (*β* = −0.00083, *P* = .02).

**Table 2:**
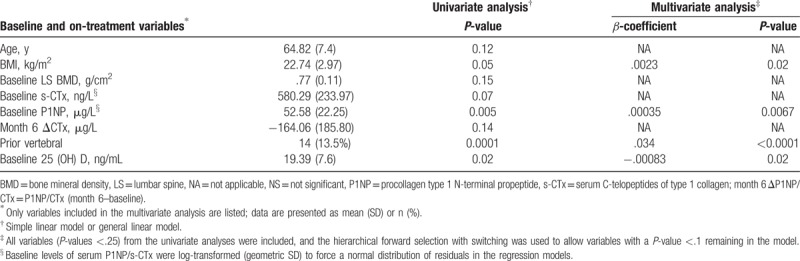
Linear modeling of characteristics associated with 1-year LS-BMD improvement in patients on calcitriol (n = 104).

## 4 Discussion

The present post hoc exploratory analysis showed that weekly 70 mg ALN/D5600 differed from daily calcitriol 0.25 μg with regard to the baseline and on-treatment characteristics that are predictive of a 1-year LS-BMD increase. Clinical characteristics including age, BMI, dietary calcium, and month-6 P1NP change were significantly associated with 1-year LS-BMD increase treated by ALN/D5600.

Osteoporosis can lead to chronic pain and fractures.^[13]^ Vitamin D alone may be controversial in the prevention of vertebral and other fractures,^[14,15]^ which is partly due to vitamin D receptor polymorphism.^[16]^ Present results further supported use of antiresorptive therapy with vitamin D and other concomitant therapies.^[17–21]^ For the identified clinical characteristics, age is a well-known risk factor of postmenopausal osteoporosis.^[22]^ Paschalis et al^[23]^ reported that aging results in both loss of bone amount and decomposition of bone material in postmenopausal women by altering the formation and maturation of trabecular bone. Imbalanced activities of osteoclasts and osteoblasts underlie postmenopausal osteoporosis due to estrogen deficiency, and bisphosphonates act to decrease bone remodeling by inhibiting osteoclast activity.^[24,25]^ Our results showed that age was independently associated with BMD improvement after ALN/D5600 treatment but not after treatment with calcitriol alone.

Body weight or BMI are variably associated with osteoporosis and fracture in postmenopausal women as it is age- and site-dependent.^[26,27]^ Low dietary calcium intake is also known to increase the risk of osteoporosis in postmenopausal women.^[28]^ Our results showed that low dietary calcium intake predicted a greater LS-BMD increase after treatment with ALN (not directly regulating calcium metabolism) plus native vitamin D but not after treatment with calcitriol alone. It remains undetermined whether postmenopausal osteoporosis women with a low dietary calcium intake benefit more from native vitamin D or its active analog as multiple factors, such as age, race, eating habits, and physical activity, may affect calcium absorption and reabsorption. Prior vertebral fracture predicts new vertebral and non-vertebral fractures probably due to the fact that these patients have more frequent and greater numbers of risk factors, such as age, concomitant medication, and baseline LS-BMD.^[29]^ Our results showed that ALN/D5600 was equally beneficial with respect to LS-BMD increase for patients with prior vertebral facture and those without, in contrast to calcitriol which had a more pronounced benefit for those with prior vertebral facture. Baseline LS-BMD is associated with BMD response at ≥2 sites of the LS, hip trochanter, femoral neck, and total hip after treatment with calcitriol,^[30]^ whereas baseline LS-BMD was independent of the 1-year increase in both the ALN/D5600 and calcitriol treatment arms.

The role of BTMs in the prediction of antiosteoporotic response has been well documented for bisphosphonates including alendronate. P1NP and s-CTx are 2 biochemical markers involved in bone formation that are often used for assessment of bone remodeling and monitoring the response to antiresorptive therapy.^[31]^ Burnett-Bowie et al^[32]^ reported that postmenopausal osteoporosis patients with the greatest on-treatment reduction in BTMs, including bone-specific alkaline phosphatase, P1NP, and s-CTx, had the greatest BMD gain as compared with nonresponders; however, baseline BTMs did not predict the 24-month BMD response. In contrast, our previous study showed that these 2 biochemical markers decrease to a significantly greater extent from baseline levels after ALN/D5600 treatment as compared with that after calcitriol treatment alone at both months 6 and 12.^[9]^ Alternatively, conventional serum biochemical markers may have a limited sensitivity and specificity for predicting the long-term BMD response; for example, urine biochemical markers have shown favorable efficiency and accuracy in determining the BMD response to antiresorptive therapy among both Eastern Asian and Caucasian postmenopausal osteoporosis patients.^[12,33,34]^ Furthermore, baseline BTMs and their on-treatment changes varied between these 2 regimens with respect to prediction of treatment response: higher baseline P1NP and s-CTX as well as greater P1NP and s-CTX decreases at month 6 were associated with a significantly greater LS-BMD improvement after ALN/D5600 treatment; however, neither baseline BTMs nor their on-treatment changes, other than baseline 25(OH)D, was associated with the LS-BMD change at 12 months after calcitriol treatment alone. It is known that 25(OH)D has a minimal benefit in osteoporosis patients without vitamin D insufficiency, whereas bisphosphonate exerts an antiresorptive effect without requiring vitamin D supplementation.^[35]^ A combination of bisphosphonate and vitamin D may have a synergistic effect in osteoporosis patients with complicating hypovitaminemia.

Vitamin D insufficiency is well known to result in poor calcium absorption, and calcitriol can effectively improve initial calcium intake.^[36]^ It remains controversial whether vitamin D insufficiency with or without low dietary calcium intake can adversely affect the BMD response after bisphosphonate treatment in postmenopausal osteoporosis patients.^[37]^ Deane et al^[38]^ reported that patients with a higher serum vitamin D (>70 nmol/L) had a greater BMD increase at the hip, and optimal vitamin D repletion may maximize treatment response of anti-absorbers in terms of BMD and fracture risk.^[39]^ For patients on alendronate treatment, a minimum level of 25(OH) above 25 ng/mL may optimize the LS-BMD response.^[40]^ Antoniucci et al^[41]^ reported that the total hip or spinal BMD response to alendronate did significantly vary among vitamin D sufficient (25(OH) >30 ng/mL), insufficient (10–30 ng/mL), and deficient (≤10 ng/mL) postmenopausal women receiving co-administration of calcitriol and calcium. Our results showed that baseline vitamin insufficiency did not affect the BMD response to alendronate in postmenopausal osteoporosis patients comedicated with vitamin D. A major confounding factor may be secondary hyperparathyroidism frequently concurring with osteoporosis in postmenopausal women.^[42]^

Although we analyzed data from a previous randomized, controlled trial with adequate statistical power and sample size, there were limitations for this study. This post hoc analysis did not predefine variables associated with antiresorptive therapy. The study aimed to display, rather than compare, potential differences in clinical characteristics associated with BMD increase between ALN/D5600 and calcitriol groups. The study analysis may be associated with an insufficient statistical power. Therefore, there might be other variables in the study that failed to be identified for ALN/D5600 versus calcitriol. Also, serum BTMs have already been known as early predictors of antiresorptive therapy.

In conclusion, the presented findings from Chinese postmenopausal osteoporotic women suggested clinically meaningful baseline and on-treatment characteristics predicting BMD improvement after 1 year of ALN/D5600 treatment, which differed from calcitriol treatment with baseline identifiable associations. Study remained exploratory and further accumulation of evidence is needed.

## Author contributions

**Conceptualization:** Er-Yuan Liao, Zhen-Lin Zhang, Wei-Bo Xia, Hua Lin, Qun Cheng, Li Wang, Yong-Qiang Hao, De-Cai Chen, Hai Tang, Yong-De Peng, Li You, Liang He, Zhao-Heng Hu, Chun-Li Song, Fang Wei, Jue Wang, Lei Zhang.

**Data curation:** Er-Yuan Liao, Zhen-Lin Zhang, Wei-Bo Xia, Hua Lin, Qun Cheng, Li Wang, Yong-Qiang Hao, De-Cai Chen, Hai Tang, Yong-De Peng, Li You, Liang He, Zhao-Heng Hu, Chun-Li Song, Fang Wei, Jue Wang, Lei Zhang.

**Formal analysis:** Er-Yuan Liao, Zhen-Lin Zhang, Wei-Bo Xia, Hua Lin, Qun Cheng, Li Wang, Yong-Qiang Hao, De-Cai Chen, Hai Tang, Yong-De Peng, Li You, Liang He, Zhao-Heng Hu, Chun-Li Song, Fang Wei, Jue Wang, Lei Zhang.

**Funding acquisition:** Er-Yuan Liao, Zhen-Lin Zhang, Wei-Bo Xia, Hua Lin, Qun Cheng, Fang Wei, Jue Wang, Lei Zhang.

**Investigation:** Er-Yuan Liao, Zhen-Lin Zhang, Wei-Bo Xia, Hua Lin, Qun Cheng, Li Wang, Yong-Qiang Hao, De-Cai Chen, Hai Tang, Yong-De Peng, Li You, Liang He, Zhao-Heng Hu, Chun-Li Song, Fang Wei, Jue Wang, Lei Zhang.

**Methodology:** Er-Yuan Liao, Zhen-Lin Zhang, Wei-Bo Xia, Hua Lin, Qun Cheng, Li Wang, Yong-Qiang Hao, De-Cai Chen, Hai Tang, Yong-De Peng, Li You, Liang He, Zhao-Heng Hu, Chun-Li Song, Fang Wei, Jue Wang, Lei Zhang.

**Project administration:** Er-Yuan Liao, Zhen-Lin Zhang, Wei-Bo Xia, Hua Lin, Qun Cheng, Li Wang, Yong-Qiang Hao, De-Cai Chen, Hai Tang, Yong-De Peng, Li You, Liang He, Chun-Li Song, Fang Wei, Jue Wang, Lei Zhang.

**Resources:** Er-Yuan Liao, Zhen-Lin Zhang, Wei-Bo Xia, Hua Lin, Qun Cheng, Li Wang, Yong-Qiang Hao, De-Cai Chen, Hai Tang, Yong-De Peng, Li You, Liang He, Zhao-Heng Hu, Chun-Li Song, Fang Wei, Jue Wang, Lei Zhang.

**Software:** Er-Yuan Liao, Zhen-Lin Zhang, Wei-Bo Xia, Hua Lin, Qun Cheng, Li Wang, Yong-Qiang Hao, De-Cai Chen, Hai Tang, Yong-De Peng, Li You, Liang He, Zhao-Heng Hu, Chun-Li Song, Fang Wei, Jue Wang, Lei Zhang.

**Supervision:** Er-Yuan Liao, Zhen-Lin Zhang, Wei-Bo Xia, Hua Lin, Qun Cheng, Fang Wei, Jue Wang, Lei Zhang.

**Validation:** Er-Yuan Liao, Zhen-Lin Zhang, Wei-Bo Xia, Hua Lin, Qun Cheng, Li Wang, Yong-Qiang Hao, De-Cai Chen, Hai Tang, Yong-De Peng, Li You, Liang He, Zhao-Heng Hu, Chun-Li Song, Fang Wei, Jue Wang, Lei Zhang.

**Visualization:** Er-Yuan Liao, Zhen-Lin Zhang, Wei-Bo Xia, Hua Lin, Qun Cheng, Li Wang, Yong-Qiang Hao, De-Cai Chen, Hai Tang, Yong-De Peng, Li You, Liang He, Zhao-Heng Hu, Chun-Li Song, Fang Wei, Jue Wang, Lei Zhang.

**Writing – original draft:** Er-Yuan Liao, Zhen-Lin Zhang, Wei-Bo Xia, Hua Lin, Qun Cheng.

**Writing – review and editing:** Er-Yuan Liao, Zhen-Lin Zhang, Wei-Bo Xia, Hua Lin, Qun Cheng, Li Wang, Yong-Qiang Hao, De-Cai Chen, Hai Tang, Yong-De Peng, Li You, Liang He, Zhao-Heng Hu, Chun-Li Song, Fang Wei, Jue Wang, Lei Zhang.

## Supplementary Material

Supplemental Digital Content

## Supplementary Material

Supplemental Digital Content
