# Supplementary material for: Clinical characteristics associated with bone mineral density improvement after 1-year alendronate/vitamin d3 or calcitriol treatment: Exploratory results from a phase 3, randomized, controlled trial on postmenopausal osteoporotic women in China
Source: Medicine (Baltimore). 2018 Aug 3;97(31):e11694. doi: 10.1097/MD.0000000000011694 (PMC6081166; doi:10.1097/MD.0000000000011694)
Supplement: Supplemental Digital Content [file medi-97-e11694-s001.pdf]

Analysis of Percentage Change of BMD at 12 Months vs. Baseline P1NP
Patients Treated with Alendronate 70 mg plus Vitamin D3 5600
(Full Analysis Set)

BMD Location	Group of Baseline P1NP	N	LS Mean	SE	95% CI
Femoral Neck (g/cm2)	Above Median	48	4.99	1.20	(2.61 - 7.38)
	Below Median	49	2.05	1.19	(-0.31 - 4.41)
Lumbar Spine (g/cm2)	Above Median	48	5.79	0.52	(4.77 - 6.82)
	Below Median	49	4.09	0.51	(3.08 - 5.11)
Total Hip (g/cm2)	Above Median	48	3.96	0.48	(3.01 - 4.91)
	Below Median	49	1.27	0.48	(0.33 - 2.21)

Longitudinal data analysis (LDA) with unstructured covariance matrix is used to model the correlation among repeated measurements. The model includes percent change of BMD from baseline as response variable, and includes terms for time, Baseline P1NP stratum (<=median versus >median), the interaction of time by Baseline P1NP stratum (<=median versus >median).
CI: Confidence intervals. SE: Standard error.
N = Number of patients with non-missing at specified group.

Analysis of Percentage Change of BMD at 12 Months vs. Baseline P1NP
Patients Treated with Alendronate 70 mg plus Vitamin D3 5600
(Full Analysis Set)

BMD Location	Comparison	Estimated Difference	SE	95% CI	p-value
Femoral Neck (g/cm2)	Above Median vs. Below Median	2.94	1.69	(-0.41 - 6.30)	0.0846
Lumbar Spine (g/cm2)	Above Median vs. Below Median	1.70	0.73	(0.26 - 3.15)	0.0216
Total Hip (g/cm2)	Above Median vs. Below Median	2.69	0.68	(1.35 - 4.03)	0.0001

Longitudinal data analysis (LDA) with unstructured covariance matrix is used to model the correlation among repeated measurements. The model includes percent change of BMD from baseline as response variable, and includes terms for time, Baseline P1NP stratum (<=median versus >median), the interaction of time by Baseline P1NP stratum (<=median versus >median).
CI: Confidence intervals. SE: Standard error.

Analysis of Percentage Change of BMD at 12 Months vs. P1NP at 12 Months
Patients Treated with Alendronate 70 mg plus Vitamin D3 5600
(Full Analysis Set)

BMD Location	Group of P1NP at 12 Months	N	LS Mean	SE	95% CI
Femoral Neck (g/cm2)	Above Median	47	4.17	1.24	(1.71 - 6.64)
	Below Median	48	2.93	1.23	(0.49 - 5.37)
Lumbar Spine (g/cm2)	Above Median	47	4.84	0.54	(3.76 - 5.92)
	Below Median	48	4.98	0.54	(3.91 - 6.04)
Total Hip (g/cm2)	Above Median	47	2.51	0.52	(1.48 - 3.54)
	Below Median	48	2.85	0.51	(1.83 - 3.86)

Longitudinal data analysis (LDA) with unstructured covariance matrix is used to model the correlation among repeated measurements. The model includes percent change of BMD from baseline as response variable, and includes terms for time, P1NP at 12 Months stratum (<=median versus >median), the interaction of time by P1NP at 12 Months stratum (<=median versus >median).
CI: Confidence intervals. SE: Standard error.
N = Number of patients with non-missing at specified group.

Analysis of Percentage Change of BMD at 12 Months vs. P1NP at 12 Months
Patients Treated with Alendronate 70 mg plus Vitamin D3 5600
(Full Analysis Set)

BMD Location	Comparison	Estimated Difference	SE	95% CI	p-value
Femoral Neck (g/cm2)	Above Median vs. Below Median	1.25	1.75	(-2.22 - 4.72)	0.4768
Lumbar Spine (g/cm2)	Above Median vs. Below Median	-0.14	0.76	(-1.65 - 1.38)	0.8571
Total Hip (g/cm2)	Above Median vs. Below Median	-0.34	0.73	(-1.78 - 1.11)	0.6447

Longitudinal data analysis (LDA) with unstructured covariance matrix is used to model the correlation among repeated measurements. The model includes percent change of BMD from baseline as response variable, and includes terms for time, P1NP at 12 Months stratum (<=median versus >median), the interaction of time by P1NP at 12 Months stratum (<=median versus >median).
CI: Confidence intervals. SE: Standard error.

Analysis of Percentage Change of BMD at 12 Months vs. Baseline s-CTx
Patients Treated with Alendronate 70 mg plus Vitamin D3 5600
(Full Analysis Set)

BMD Location	Group of Baseline s-CTx	N	LS Mean	SE	95% CI
Femoral Neck (g/cm2)	Above Median	47	4.39	1.23	(1.95 - 6.82)
	Below Median	50	2.68	1.19	(0.32 - 5.04)
Lumbar Spine (g/cm2)	Above Median	47	5.18	0.54	(4.12 - 6.25)
	Below Median	50	4.70	0.52	(3.67 - 5.73)
Total Hip (g/cm2)	Above Median	47	3.33	0.51	(2.31 - 4.35)
	Below Median	50	1.91	0.50	(0.93 - 2.90)

Longitudinal data analysis (LDA) with unstructured covariance matrix is used to model the correlation among repeated measurements. The model includes percent change of BMD from baseline as response variable, and includes terms for time, Baseline s-CTx stratum (<=median versus >median), the interaction of time by Baseline s-CTx stratum (<=median versus >median).
CI: Confidence intervals. SE: Standard error.
N = Number of patients with non-missing at specified group.

Analysis of Percentage Change of BMD at 12 Months vs. Baseline s-CTx
Patients Treated with Alendronate 70 mg plus Vitamin D3 5600
(Full Analysis Set)

BMD Location	Comparison	Estimated Difference	SE	95% CI	p-value
Femoral Neck (g/cm2)	Above Median vs. Below Median	1.71	1.71	(-1.68 - 5.10)	0.3191
Lumbar Spine (g/cm2)	Above Median vs. Below Median	0.48	0.75	(-1.00 - 1.97)	0.5180
Total Hip (g/cm2)	Above Median vs. Below Median	1.42	0.72	(-0.00 - 2.84)	0.0507

Longitudinal data analysis (LDA) with unstructured covariance matrix is used to model the correlation among repeated measurements. The model includes percent change of BMD from baseline as response variable, and includes terms for time, Baseline s-CTx stratum (<=median versus >median), the interaction of time by Baseline s-CTx stratum (<=median versus >median).
CI: Confidence intervals. SE: Standard error.

Analysis of Percentage Change of BMD at 12 Months vs. s-CTx at 12 Months
Patients Treated with Alendronate 70 mg plus Vitamin D3 5600
(Full Analysis Set)

BMD Location	Group of s-CTx at 12 Months	N	LS Mean	SE	95% CI
Femoral Neck (g/cm2)	Above Median	47	3.82	1.24	(1.35 - 6.29)
	Below Median	48	3.27	1.23	(0.82 - 5.71)
Lumbar Spine (g/cm2)	Above Median	47	4.74	0.54	(3.66 - 5.81)
	Below Median	48	5.08	0.54	(4.01 - 6.14)
Total Hip (g/cm2)	Above Median	47	2.91	0.52	(1.88 - 3.93)
	Below Median	48	2.45	0.51	(1.44 - 3.47)

Longitudinal data analysis (LDA) with unstructured covariance matrix is used to model the correlation among repeated measurements. The model includes percent change of BMD from baseline as response variable, and includes terms for time, s-CTx at 12 Months stratum (<=median versus >median), the interaction of time by s-CTx at 12 Months stratum
(<=median versus >median).
CI: Confidence intervals. SE: Standard error.
N = Number of patients with non-missing at specified group.

Analysis of Percentage Change of BMD at 12 Months vs. s-CTx at 12 Months
Patients Treated with Alendronate 70 mg plus Vitamin D3 5600
(Full Analysis Set)

BMD Location	Comparison	Estimated Difference	SE	95% CI	p-value
Femoral Neck (g/cm2)	Above Median vs. Below Median	0.55	1.75	(-2.92 - 4.03)	0.7523
Lumbar Spine (g/cm2)	Above Median vs. Below Median	-0.34	0.76	(-1.85 - 1.18)	0.6570
Total Hip (g/cm2)	Above Median vs. Below Median	0.45	0.73	(-0.99 - 1.90)	0.5359

Longitudinal data analysis (LDA) with unstructured covariance matrix is used to model the correlation among repeated measurements. The model includes percent change of BMD from baseline as response variable, and includes terms for time, s-CTx at 12 Months stratum (<=median versus >median), the interaction of time by s-CTx at 12 Months stratum
(<=median versus >median).
CI: Confidence intervals. SE: Standard error.
