# Supplementary material for: Clinical characteristics associated with bone mineral density improvement after 1-year alendronate/vitamin d3 or calcitriol treatment: Exploratory results from a phase 3, randomized, controlled trial on postmenopausal osteoporotic women in China
Source: Medicine (Baltimore). 2018 Aug 3;97(31):e11694. doi: 10.1097/MD.0000000000011694 (PMC6081166; doi:10.1097/MD.0000000000011694)
Supplement: Supplemental Digital Content [file medi-97-e11694-s002.pdf]

Analysis of Percentage Change of BMD at 12 Months vs. Tertiles of Baseline 25(OH)D
Patients Treated with Alendronate 70 mg plus Vitamin D3 5600
(Full Analysis Set)

BMD Location	Tertiles Group of Baseline 25(OH)D	N	LS Mean	SE	95% CI
Femoral Neck (g/cm2)	First Tertile	29	3.41	1.58	(0.28 - 6.54)
	Second Tertile	36	3.38	1.42	(0.57 - 6.19)
	Third Tertile	32	3.74	1.50	(0.76 - 6.72)
Lumbar Spine (g/cm2)	First Tertile	29	5.34	0.69	(3.98 - 6.70)
	Second Tertile	36	5.00	0.62	(3.78 - 6.22)
	Third Tertile	32	4.49	0.65	(3.20 - 5.79)
Total Hip (g/cm2)	First Tertile	29	3.15	0.66	(1.84 - 4.46)
	Second Tertile	36	1.76	0.59	(0.58 - 2.94)
	Third Tertile	32	3.05	0.63	(1.81 - 4.30)

Longitudinal data analysis (LDA) with unstructured covariance matrix is used to model the correlation among repeated measurements. The model includes percent change of BMD from baseline as response variable, and includes terms for time, tertiles of Baseline 25(OH)D, the interaction of time by tertiles of baseline 25(OH)D.
CI: Confidence intervals. SE: Standard error.
N = Number of patients with non-missing at specified group.

Analysis of Percentage Change of BMD at 12 Months vs. Tertiles of Baseline 25(OH)D
Patients Treated with Alendronate 70 mg plus Vitamin D3 5600
(Full Analysis Set)

BMD Location	Comparison	Estimated Difference	SE	95% CI	p-value
Femoral Neck (g/cm2)	First Tertile vs. Second Tertile	0.03	2.12	(-4.18 - 4.23)	0.9900
	First Tertile vs. Third Tertile	-0.33	2.18	(-4.65 - 3.99)	0.8804
	Second Tertile vs. Third Tertile	-0.36	2.06	(-4.45 - 3.74)	0.8637
Lumbar Spine (g/cm2)	First Tertile vs. Second Tertile	0.34	0.92	(-1.48 - 2.17)	0.7089
	First Tertile vs. Third Tertile	0.85	0.95	(-1.03 - 2.73)	0.3726
	Second Tertile vs. Third Tertile	0.50	0.90	(-1.28 - 2.28)	0.5762
Total Hip (g/cm2)	First Tertile vs. Second Tertile	1.39	0.89	(-0.37 - 3.15)	0.1213
	First Tertile vs. Third Tertile	0.09	0.91	(-1.72 - 1.90)	0.9200
	Second Tertile vs. Third Tertile	-1.29	0.86	(-3.01 - 0.42)	0.1371

Longitudinal data analysis (LDA) with unstructured covariance matrix is used to model the correlation among repeated measurements. The model includes percent change of BMD from baseline as response variable, and includes terms for
time, tertiles of Baseline 25(OH)D, the interaction of time by tertiles of baseline 25(OH)D.
CI: Confidence intervals. SE: Standard error.

Analysis of Percentage Change of BMD at 12 Months vs. Tertiles of 25(OH)D at 12 Months
Patients Treated with Alendronate 70 mg plus Vitamin D3 5600
(Full Analysis Set)

BMD Location	Tertiles Group of 25(OH)D at 12 Months	N	LS Mean	SE	95% CI
Femoral Neck (g/cm2)	First Tertile	29	5.51	1.57	(2.39 - 8.64)
	Second Tertile	39	2.19	1.35	(-0.50 - 4.88)
	Third Tertile	27	3.38	1.63	(0.15 - 6.61)
Lumbar Spine (g/cm2)	First Tertile	29	5.75	0.69	(4.38 - 7.11)
	Second Tertile	39	4.55	0.59	(3.37 - 5.73)
	Third Tertile	27	4.53	0.71	(3.12 - 5.95)
Total Hip (g/cm2)	First Tertile	29	2.94	0.66	(1.64 - 4.25)
	Second Tertile	39	3.00	0.57	(1.87 - 4.12)
	Third Tertile	27	1.93	0.68	(0.58 - 3.28)

Longitudinal data analysis (LDA) with unstructured covariance matrix is used to model the correlation among repeated measurements. The model includes percent change of BMD from baseline as response variable, and includes terms for time, tertiles of 25(OH)D at 12 Months, the interaction of time by tertiles of baseline 25(OH)D.
CI: Confidence intervals. SE: Standard error.
N = Number of patients with non-missing at specified group.

Analysis of Percentage Change of BMD at 12 Months vs. Tertiles of 25(OH)D at 12 Months
Patients Treated with Alendronate 70 mg plus Vitamin D3 5600
(Full Analysis Set)

BMD Location	Comparison	Estimated Difference	SE	95% CI	p-value
Femoral Neck (g/cm2)	First Tertile vs. Second Tertile	3.32	2.07	(-0.80 - 7.45)	0.1125
	First Tertile vs. Third Tertile	2.13	2.26	(-2.36 - 6.63)	0.3481
	Second Tertile vs. Third Tertile	-1.19	2.12	(-5.40 - 3.02)	0.5755
Lumbar Spine (g/cm2)	First Tertile vs. Second Tertile	1.20	0.91	(-0.60 - 3.00)	0.1898
	First Tertile vs. Third Tertile	1.22	0.99	(-0.75 - 3.18)	0.2221
	Second Tertile vs. Third Tertile	0.02	0.93	(-1.82 - 1.86)	0.9848
Total Hip (g/cm2)	First Tertile vs. Second Tertile	-0.05	0.87	(-1.78 - 1.67)	0.9498
	First Tertile vs. Third Tertile	1.01	0.95	(-0.87 - 2.89)	0.2872
	Second Tertile vs. Third Tertile	1.07	0.89	(-0.69 - 2.83)	0.2312

Longitudinal data analysis (LDA) with unstructured covariance matrix is used to model the correlation among repeated measurements. The model includes percent change of BMD from baseline as response variable, and includes terms for
time, tertiles of 25(OH)D at 12 Months, the interaction of time by tertiles of baseline 25(OH)D.
CI: Confidence intervals. SE: Standard error.

Analysis of Percentage Change of BMD at 12 Months vs. Tertiles of 25(OH)D Absolute Change at 12 Months
Patients Treated with Alendronate 70 mg plus Vitamin D3 5600
(Full Analysis Set)

BMD Location	Tertiles Group of 25(OH)D Absolute Change at 12 Months	N	LS Mean	SE	95% CI
Femoral Neck (g/cm2)	First Tertile	30	3.80	1.57	(0.69 - 6.91)
	Second Tertile	36	3.32	1.43	(0.48 - 6.16)
	Third Tertile	29	3.56	1.59	(0.39 - 6.72)
Lumbar Spine (g/cm2)	First Tertile	30	4.64	0.67	(3.31 - 5.97)
	Second Tertile	36	5.74	0.61	(4.52 - 6.96)
	Third Tertile	29	4.16	0.68	(2.80 - 5.52)
Total Hip (g/cm2)	First Tertile	30	2.92	0.65	(1.63 - 4.21)
	Second Tertile	36	2.92	0.59	(1.74 - 4.10)
	Third Tertile	29	2.13	0.66	(0.82 - 3.44)

Longitudinal data analysis (LDA) with unstructured covariance matrix is used to model the correlation among repeated measurements. The model includes percent change of BMD from baseline as response variable, and includes terms for time, tertiles of 25(OH)D Absolute Change at 12 Months, the interaction of time by tertiles of baseline 25(OH)D.
CI: Confidence intervals. SE: Standard error.
N = Number of patients with non-missing at specified group.

Analysis of Percentage Change of BMD at 12 Months vs. Tertiles of 25(OH)D Absolute Change at 12 Months
Patients Treated with Alendronate 70 mg plus Vitamin D3 5600
(Full Analysis Set)

BMD Location	Comparison	Estimated Difference	SE	95% CI	p-value
Femoral Neck (g/cm2)	First Tertile vs. Second Tertile	0.47	2.12	(-3.74 - 4.68)	0.8242
	First Tertile vs. Third Tertile	0.24	2.23	(-4.20 - 4.68)	0.9146
	Second Tertile vs. Third Tertile	-0.23	2.14	(-4.48 - 4.02)	0.9138
Lumbar Spine (g/cm2)	First Tertile vs. Second Tertile	-1.10	0.91	(-2.90 - 0.71)	0.2307
	First Tertile vs. Third Tertile	0.48	0.96	(-1.42 - 2.38)	0.6158
	Second Tertile vs. Third Tertile	1.58	0.92	(-0.24 - 3.40)	0.0886
Total Hip (g/cm2)	First Tertile vs. Second Tertile	-0.00	0.88	(-1.75 - 1.74)	0.9990
	First Tertile vs. Third Tertile	0.79	0.93	(-1.05 - 2.63)	0.3960
	Second Tertile vs. Third Tertile	0.79	0.89	(-0.97 - 2.55)	0.3751

Longitudinal data analysis (LDA) with unstructured covariance matrix is used to model the correlation among repeated measurements. The model includes percent change of BMD from baseline as response variable, and includes terms for
time, tertiles of 25(OH)D Absolute Change at 12 Months, the interaction of time by tertiles of baseline 25(OH)D.
CI: Confidence intervals. SE: Standard error.
